# Animal survival strategies in Neoproterozoic ice worlds

**DOI:** 10.1111/gcb.16393

**Published:** 2022-10-11

**Authors:** Huw J. Griffiths, Rowan J. Whittle, Emily G. Mitchell

**Affiliations:** ^1^ British Antarctic Survey Cambridge UK; ^2^ Department of Zoology University of Cambridge Cambridge UK

**Keywords:** Cryogenian, Ediacaran, Gaskiers, Marinoan, polar, slushball Earth, snowball Earth, Sturtian

## Abstract

The timing of the first appearance of animals is of crucial importance for understanding the evolution of life on Earth. Although the fossil record places the earliest metazoans at 572–602 Ma, molecular clock studies suggest a far earlier origination, as far back as ~850 Ma. The difference in these dates would place the rise of animal life into a time period punctuated by multiple colossal, potentially global, glacial events. Although the two schools of thought debate the limitations of each other's methods, little time has been dedicated to how animal life might have survived if it did arise before or during these global glacial periods. The history of recent polar biota shows that organisms have found ways of persisting on and around the ice of the Antarctic continent throughout the Last Glacial Maximum (33–14 Ka), with some endemic species present before the breakup of Gondwana (180–23 Ma). Here we discuss the survival strategies and habitats of modern polar marine organisms in environments analogous to those that could have existed during Neoproterozoic glaciations. We discuss how, despite the apparent harshness of many ice covered, sub‐zero, Antarctic marine habitats, animal life thrives on, in and under the ice. Ice dominated systems and processes make some local environments more habitable through water circulation, oxygenation, terrigenous nutrient input and novel habitats. We consider how the physical conditions of Neoproterozoic glaciations would likely have dramatically impacted conditions for potential life in the shallows and erased any possible fossil evidence from the continental shelves. The recent glacial cycle has driven the evolution of Antarctica's unique fauna by acting as a “diversity pump,” and the same could be true for the late Proterozoic and the evolution of animal life on Earth, and the existence of life elsewhere in the universe on icy worlds or moons.

## Graphical abstract

The fossil record places the earliest metazoans at 572–602 Ma, whilst molecular clock studies suggest an earlier origination, up to ~850 Ma, during a time punctuated by multiple global glacial events (snowball and slushball Earths). If animal life did arise before or during these glacial periods it would have faced conditions analogous with modern polar marine habitats, requiring similar survival strategies. Ice dominated systems make some environments more habitable and recent glacial cycles have driven the evolution of Antarctica’s unique fauna, the same could be true for the late Proterozoic and the evolution of animal life on Earth.

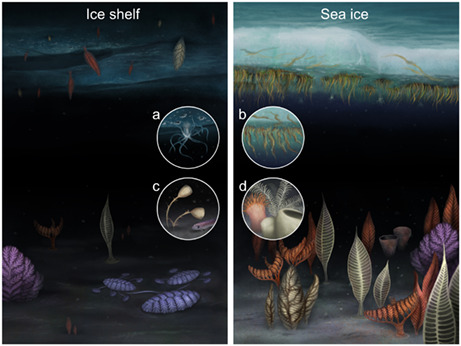

## 1 INTRODUCTION

**FIGURE 1:**
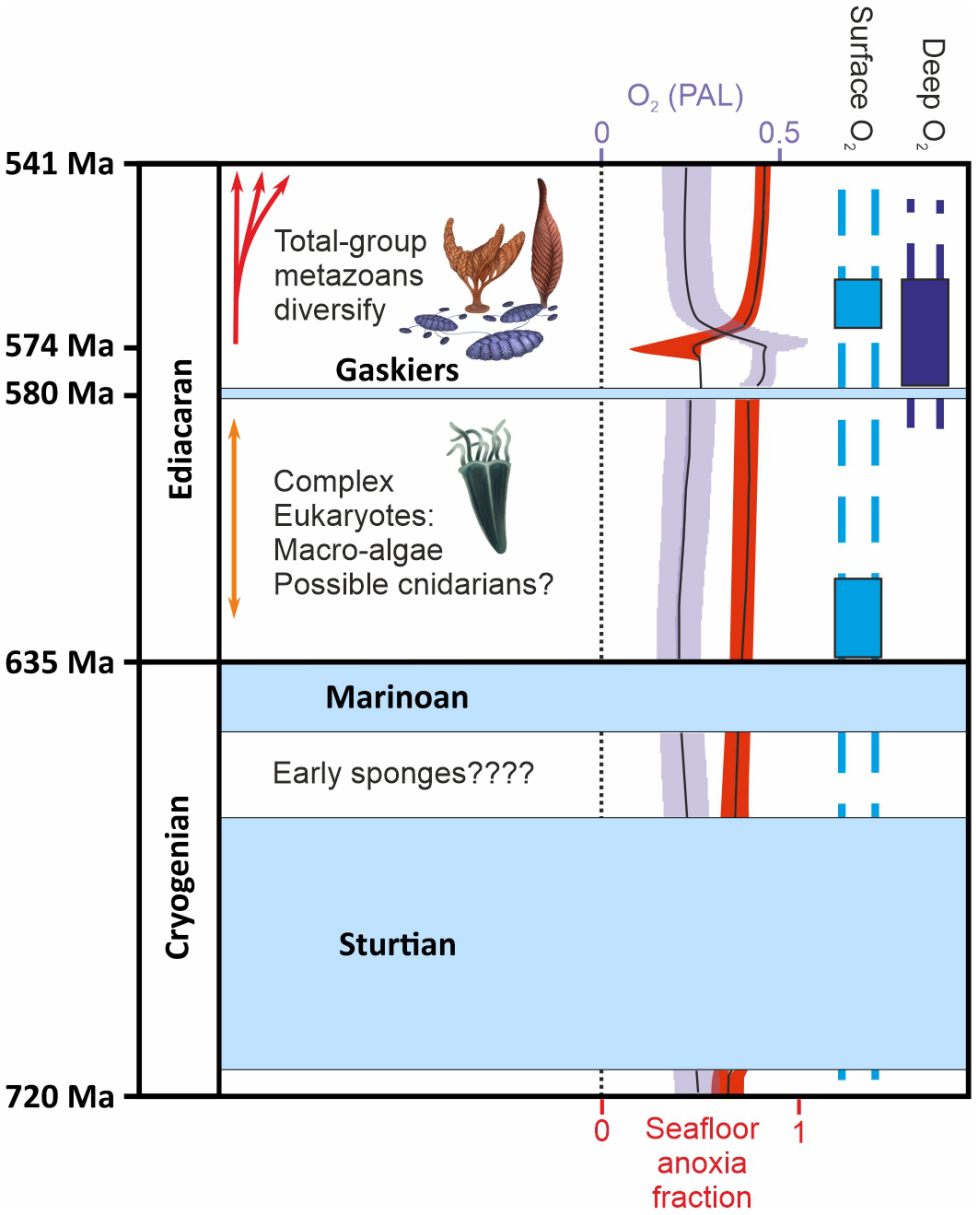
Timeline showing the three major glaciations of the Neoproterozoic (in light blue), first appearances of complex, macroscopic eukaryotes, modelled atmospheric oxygen levels (in violet), modelled seafloor anoxia (in red) (from Tostevin & Mills, 2020), and surface (blue) and deep (purple) ocean oxygen events (from Shields‐Zhou & Och, 2011).

Stem‐group eumetazoans are first found widespread in the fossil record around 572–602 Ma (Budd & Jensen, 2017; Dunn et al., 2021; Wood et al., 2019; Yang et al., 2022), yet recent molecular clock studies suggest a much earlier metazoan origination time of around 800–850 Ma (Beavan et al., 2021; Dohrmann & Wörheide, 2017; dos Reis et al., 2015; Maria Costa‐Paiva et al., 2022; Figure 1). Given the inherent patchiness of the fossil record and the uncertainties of molecular clock estimates (Budd & Mann, 2020; dos Reis et al., 2015) the exact timing of this key evolutionary milestone is hard to resolve. The recent discovery of a putative fossil keratose sponge (~890 Ma) (Turner, 2021) and the molecular dating of the blood pigment hemocyanin to 881 Ma would push back the inception of animals by around 200 million years (Maria Costa‐Paiva et al., 2022). Even the most conservative molecular clock estimates push the origins of animal life to the edge of the Ediacaran Gaskiers glaciation (a so‐called slushball Earth ~580 Ma), whereas estimates beyond 720 Ma would require life to have this survived two further global glaciation events (Marinoan and Sturtian snowball Earths) during the Cryogenian (Peterson et al., 2004).

The glaciation events of the Neoproterozoic represented a significant challenge even to the simplest of lifeforms. The Cryogenian (720–635 Ma) was dominated by two vast and long‐lasting global glaciations with widespread ice at the equator, or snowball Earths (Zhou et al., 2019). The first Cryogenian snowball Earth, the Sturtian glaciation, lasted for the first 50–60 million years of the period, and the Marinoan for the final 15 million years (Hoffmann et al., 2004; Macdonald et al., 2010). The later Gaskiers glaciation, described as a slushball Earth, was a smaller scale glaciation event which occurred during the Ediacaran (635–541 Ma). It was far shorter than the two earlier snowball Earths, lasting under 340 thousand years (Pu et al., 2016). The Gaskiers slushball glaciation was less widespread than the Sturtian or Marinoan snowball Earths, with polar caps suggested to have reached low latitudes (~30°), constituting a partial rather than full global glaciation, where a water belt of open ocean remained around the equator (Pu et al., 2016), with landmasses and shallow water spanning both the equatorial open water belt (<30° S/N) and glacial zones (>30° S/N) (Merdith et al., 2020).

Although the idea that life persisted and even diversified during global icehouse periods is under ongoing debate (Beavan et al., 2021; Budd & Mann, 2020; Nettersheim et al., 2019), and it has been suggested that more favorable, less glacially impacted, habitats might have existed (Hoffman et al., 2017), fewer studies have been dedicated to how animals, if they did exist, might have survived these most hostile periods in the history of life on Earth in among the ice. This paper uses modern‐day polar habitats and organisms as analogues, to explore types of marine and aquatic habitats that could have existed during the global glacial events of the Neoproterozoic (720–541 Ma) and where (if it existed) animal life might have survived. We also examine how glacial processes like those in Antarctica during and since the LGM may have contributed to the macroevolutionary patterns we see during the Neoproterozoic.

The Quaternary and recent history of Antarctica includes marine, freshwater, and terrestrial organisms that have all persisted in the region since before the Last Glacial Maximum (LGM), with some endemic species present since before the breakup of Gondwana (Convey et al., 2008; Lau et al., 2020). Modern and recent polar faunas provide useful insights into survival strategies in habitats analogous to those that potentially existed during Neoproterozoic glaciations. The concept of the potential survival of animal life under snowball or slushball Earth conditions has far reaching implications with regard to the possibility of life existing and surviving beyond the Earth on frozen moons, such as Jupiter's Europa, Ganymede and Callisto, and planets, where liquid water flows beneath the icy surface.

## 2 POLAR ANALOGUES

**FIGURE 2:**
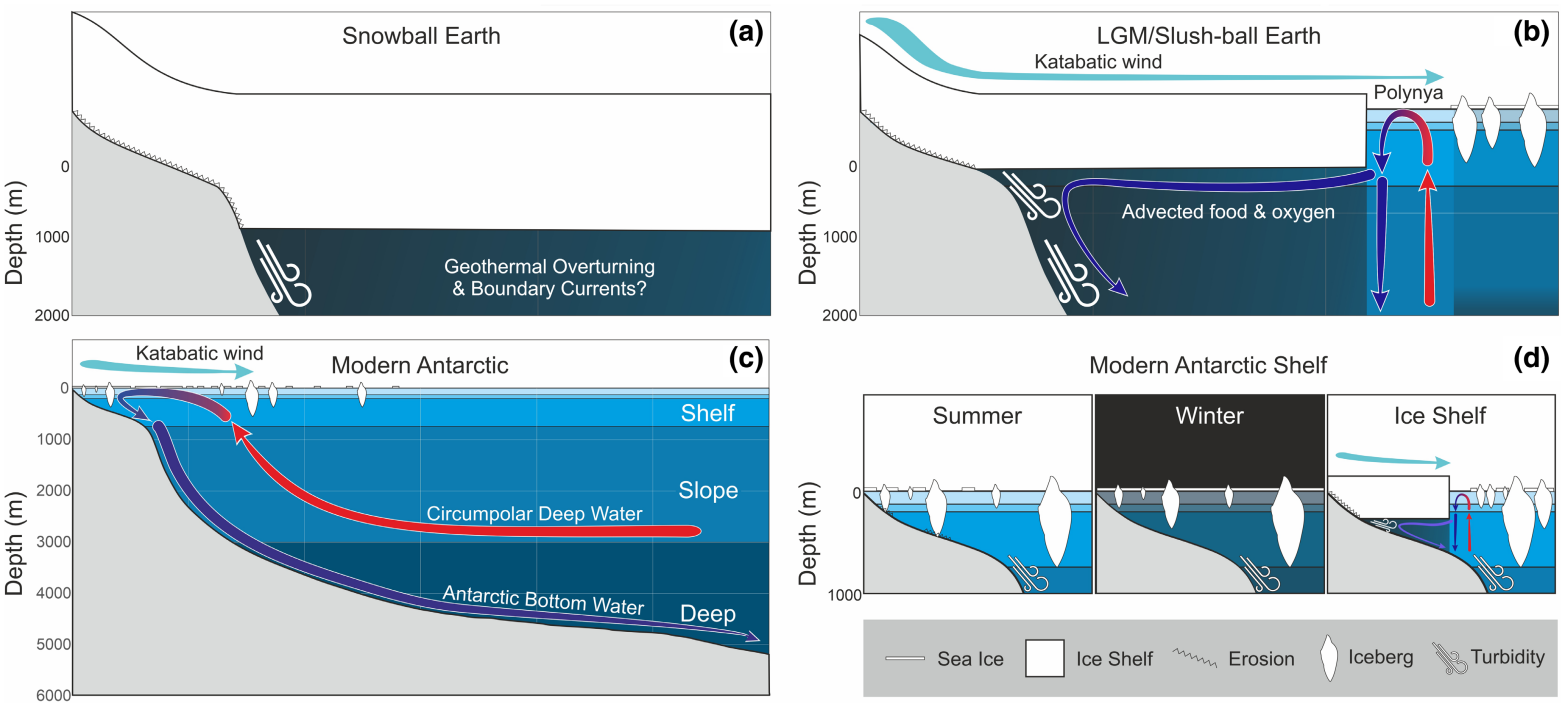
Interactions between the cryosphere and marine realm during (a) snowball Earth and (b) slushball Earth or Last Glacial Maximum Antarctica, compared with modern‐day Antarctic (c) water mass circulation and the formation of Antarctic bottom water and (d) seasonal and regional disturbance and variability on the shelf. Katabatic winds carry cold high‐density air from the continent northwards. Polynyas are areas of open water surrounded by sea ice, often produced by katabatic winds.

Modern‐day polar regions provide our best analogue for extrapolating how animal life could survive protracted glaciations. The evolution of modern marine life in the polar regions has been driven by massive upheaval from the push and pull of glacial and interglacial periods, forcing animals to adapt, diversify and specialise in a frozen world (Clarke & Crame, 1992). The Arctic is an ocean surrounded by land and was covered by thick ice sheets as recently as 60 k years ago (Geibert et al., 2021). In contrast, Antarctica is a continent entombed under an average of 2 km of ice that has been heavily glaciated for around 15 million years (Convey et al., 2008). In addition to the ice on land, Antarctica has huge floating ice shelves covering around one third of its continental shelf and the icy outline of the continent roughly doubles in area every winter as the sea surface around it freezes (Figure 2; Gloersen, 1992; Griffiths et al., 2021). The grounded ice sheets, floating ice shelves, icebergs and mobile and seasonal sea ice all provide glimpses of habitats that would have existed during snowball and slushball Neoproterozoic glacial events. Given that 90% of all the world's ice is found in Antarctica, we have largely used Antarctic and Southern Ocean examples of habitats and survival strategies as they represent stronger analogues to snowball or slushball Earth conditions than the present‐day Arctic.

The terrestrial polar realm is host to a wide range of extreme habitats, such as Antarctica's Dry Valleys, glaciers, snow, cryoconite holes, meltwater ponds, subglacial lakes, and permafrost, which are known to support microbial and more complex life (Lee et al., 2022; Shain et al., 2021; Siegert et al., 2001; Solon et al., 2021; Zawierucha et al., 2015). The global pre‐Phanerozoic fossil record is dominated by marine rather than terrestrial fossils (Wood et al., 2019). Nonetheless, key non‐marine discoveries record possible holozoans at 1 Ga (Strother et al., 2021) and fungus‐like microfossils at ~635 Ma (Gan et al., 2021). However, there are no records of Neoproterozoic terrestrial macrofossils (Xiao et al., 2013), so that while the terrestrial environment and ice may have provided refugia for complex microbial life, it is unlikely to have aided the survival of early animals.

### 2.1 Sea ice

The Antarctic sea ice is a larger physical habitat than the continent, with peaks in area of ~18 million km^2^ during September compared to the 14.2 million km^2^ of the continent (Turner et al., 2017). The majority of this ice melts during the summer, leaving around 3 million km^2^ of multiyear sea ice (Gloersen, 1992). This freeze and thaw cycle of the Antarctic sea ice provides seasonal and semi‐permanent habitats for marine life under, on and within the ice. The sea ice is usually between 1 and 2‐m thick, thus while reducing light to the water beneath it does not completely block it, and photosynthesisers survive and thrive in brine channels and on the underside of the ice (Leeuwe et al., 2020; Murray et al., 2012; Smith et al., 2012; Syvertsen, 1991). Arctic and Antarctic sea ice provides a physical framework supporting around 1000 species of single‐celled eukaryotes (van Leeuwe et al., 2018), which in turn feed small invertebrates and even larval and juvenile fish (Kohlbach et al., 2016).

The Gaskiers glacial event was much less severe than the Sturtian or Marinoan, with a water belt of open ocean around the equator, and the polar ice sheets, shelves and sea ice reaching low latitudes (>30° S/N) rather than a global coverage (Pu et al., 2016; Merdith et al., 2020). Unlike the Marinoan and Sturtian snowball Earths, the partial glaciation of the Gaskiers, would have had a gradient of seasonally affected sea ice and polynyas (areas of open water surrounded by sea ice) around the margins of any ice shelves (Fairchild & Kennedy, 2007), as we see in modern Antarctica (Figure 2).

**FIGURE 3:**
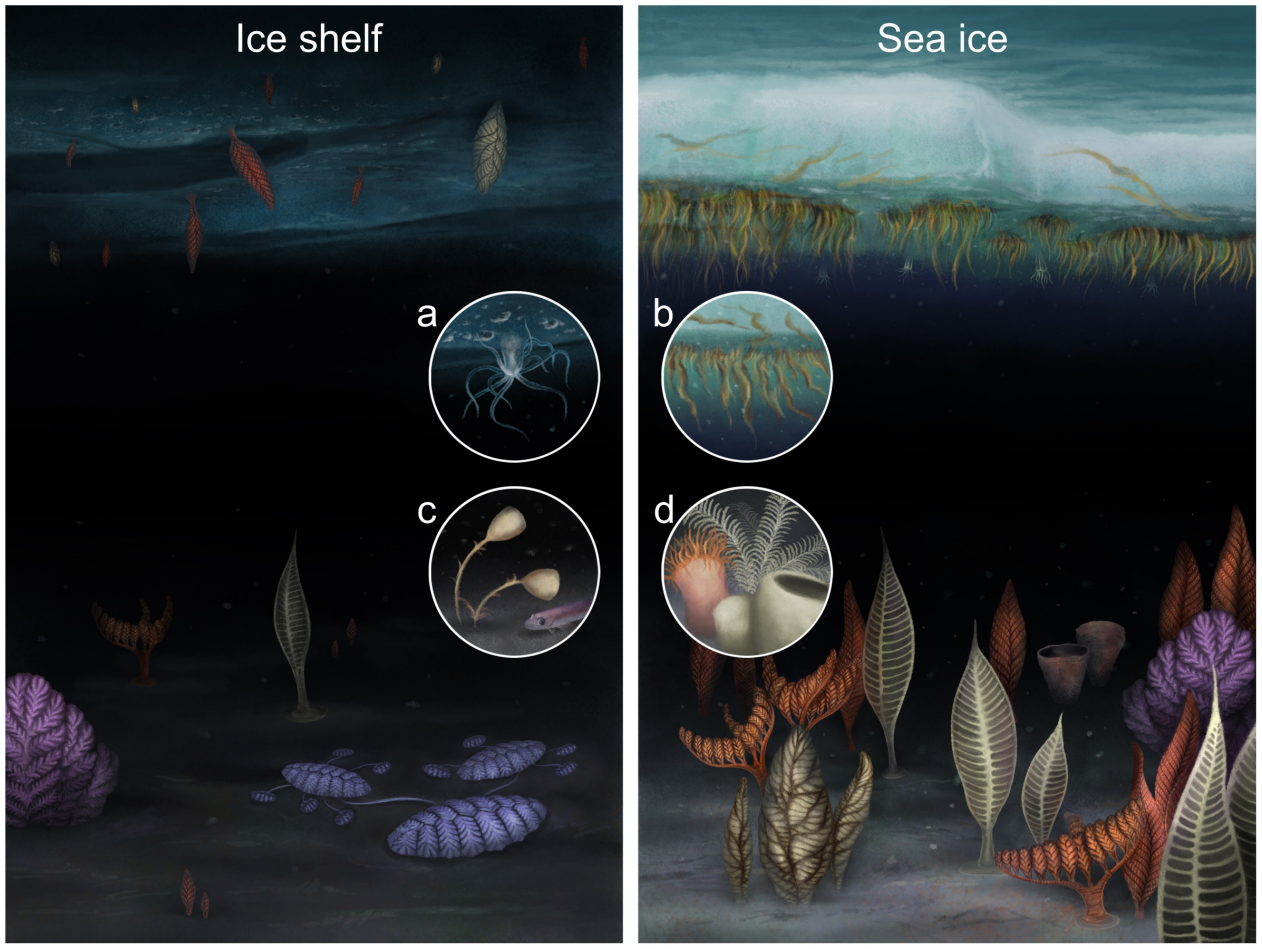
Reconstruction of possible Cryogenian or Ediacaran life under an ice shelf (left) and under Ediacaran Sea ice (right). Inset images represent modern‐day analogues: (a) Edwardsiella andrillae on the underside of the Ross Sea ice, (b) strands of the diatom Melosira arctica under Arctic Sea ice, (c) stalked sponges and a fish representing life observed under Antarctic ice shelves, and (d) sponges, anemones, and crinoids representing a typical Antarctic sub‐sea‐ice benthic communities. Reconstruction by Franz Anthony.

During the Gaskiers slushball glaciation, sea ice would have provided key habitats and resources, potentially covering up to ~40% of the Earth's surface (200 million km^2^). Today, the melting of sea ice in spring and early summer seeds the water with microalgae and microbes (van Leeuwe et al., 2018) from within the ice, leading to rapid and extensive blooms (Fillinger et al., 2013; Smith et al., 2012). These blooms result in gravitational (vertical) as well as advected food sources which feed zooplankton and the benthos (Kohlbach et al., 2016). In addition, clumps of oxygen producing algae that form on the underside of sea ice (Bernard et al., 2019) (Figure 3b), sink rapidly to the ocean floor providing high‐quality food to benthic communities below (Figure 3d).

The underside of sea ice can also be considered an upside down shallow benthic habitat with normally planktonic species such as the Antarctic krill (*Euphausia superba*) and the ice or crystal krill (*Euphausia crystallorophias*) feeding on bacteria, diatoms, detritus, and other microorganisms, including the algae that form on the underside of sea ice (Bernard et al., 2019). Sea ice also provides physical substrate for some benthic organisms such as the anemone *Edwardsiella andrillae* (Daly et al., 2013) and antarcturid isopods (Bornemann et al., 2016), living upside down attached to the ice instead of the sea floor (Figure 3a,b).

Similarly, during the Gaskiers partial glaciation, the large extent of sea ice would have increased the hard‐substrate available for benthic animals and filamentous algae (as with the modern filamentous diatom *Melosira arctica* under Arctic sea ice [van Leeuwe et al., 2018]). As such, the sea ice may have aided the survival of algae, preserved from ~900 Ma (Maloney et al., 2021) and enabled their widespread distribution, along with early animals (Wang et al., 2014; Yuan et al., 2013), immediately post‐Gaskiers (Boddy et al., 2022). These algae could have also contributed a valuable food source and oxygen to heterotrophic benthic communities underneath the sea ice and on the seafloor below (Figure 3).

### 2.2 Ice shelves

Ice shelves are huge slabs of floating glacial ice that have been pushed off the land by the glaciers and ice streams that feed them (Figure 2). The majority of ice shelves are found in Antarctica, with a few smaller ones in the Arctic (Griffiths et al., 2021). Ice shelves cover around a third of Antarctica's continental shelf, an area of around 1.6 million km^2^, and surround 75% of the coastline (Paolo et al., 2015). The thickness of ice shelves can range from about 100 m to a kilometre (Griggs & Bamber, 2011) and the largest ice shelf covers an area of 50,000 km^2^ (Rignot et al., 2013). To all intents and purposes, the area under these ice shelves can be compared to a giant cave ecosystem, largely dependent on food advected in from more open water, due to the thickness of the ice preventing in situ photosynthesis (Griffiths et al., 2021).

During the Marinoan and Sturtian global glaciations it is estimated that the global ocean would have been covered by a layer of ice around 1 km thick, like the largest of the floating ice shelves found in Antarctica today (Ashkenazy et al., 2014). Models suggest that strong jets and meridional overturning would have existed beneath the ice at the equator, driven by geothermal heat (Ashkenazy et al., 2014). Three‐dimensional ocean models, featuring reconstructed continental configurations, show further boundary currents, strong upwelling and downwelling near the continents (Ashkenazy et al., 2014). Similarly, tidally active liquid oceans survive under thick ice layer on extra‐terrestrial bodies such as Enceladus, a small, geologically active ice moon of Saturn (Roberts & Nimmo, 2008). Under a fully glaciated Earth, any liquid seawater potentially reached temperatures as low as between −2.7°C (similar to Antarctica today) and −4.7°C, depending on salinity and latitude (Ashkenazy et al., 2014). Despite the cold temperatures and ice cover, microfossil evidence suggests that phytoplankton were present before and during these glacial periods and that enhanced marine photosynthesis likely occurred throughout this period (Martin et al., 2008).

When clear ice cover exceeds 20 m thickness sub‐ice phototrophy ceases (Hoffman et al., 2017; Warren et al., 2002). Modelled solutions to snowball Earth predict that only the tropics were likely to have sufficiently thin ice to permit this (McKay, 2000). However, indirect geochemical and microfossil evidence and fossils of crown groups of several marine algae prior to 750 Ma, suggests that marine primary productivity, and life in general, must have continued throughout the Sturtian and Marinoan snowball glaciations, suggesting that there were likely swathes of thinner ice or even patches of open water (Corsetti et al., 2006; Johnson et al., 2017; Martin et al., 2008; Narbonne, 2005; Olcott et al., 2005).

Even in this seemingly most oligotrophic of marine environments life has been found with under‐ice benthic communities consuming food, such as phytoplankton and detritus that are transported by advected currents from the open water to underneath the ice (Figure 3c). The furthest that life has been found from daylight anywhere in the world is the Ross Ice Shelf, Antarctica, with amphipods, fish, jellyfish, and ctenophores observed 700 km from the nearest open water (Bruchhausen et al., 1979; Lipps et al., 1979; Stevens et al., 2020). These organisms, and those from seven other boreholes suggested that the animal life found hundreds of kilometres under ice shelves were mobile scavengers and predators, simply following food brought in by ocean currents. However, recent images from the Filchner‐Ronne Ice Shelf challenged the paradigm that sessile organisms reduce in prevalence with distance from open water, with the discovery of a boulder providing a hard substrate for an established community consisting of only sessile, likely filter feeding, organisms including sponges, 260 km from the ice front (Griffiths et al., 2021). If the direction of the prevailing currents are taken into account then the nearest food source for this community is actually somewhere between 625 km and 1500 km.

These sub‐ice shelf habitats superficially resemble a snowball Earth, with 1 km thick ice capping the ocean surface, but are closer to a scaled down partial glaciation or slushball Earth, with a dynamic oceanographic connection to the sea ice zone. While pelagic and mobile benthic animals had yet to evolve by the Gaskiers slushball glaciation (Evans et al., 2019), we suggest that the seafloor underneath ice shelves could have provided a habitat for benthic animal communities, relying on food and oxygen advected long distances from open water or polynyas along the ice shelf margin (Figures 2 and 3). A full glaciated Earth would likely only have a very limited thin ice zone as a main source of primary production (McKay, 2000). Recent work on sub‐ice shelf microbial communities in the Ross Sea found abundant, distinct and highly adapted communities dominated by aerobic lithoautotrophic archaea and bacteria capable of using ammonium, nitrite, and sulphur compounds and aerobic organoheterotrophic bacteria that can breakdown potentially advected organic carbon (Martínez‐Pérez et al., 2022). Such specialised chemosynthetic microbial communities could provide a food source for heterotrophic organisms.

Other chemosynthetic habitats offer an alternative source of food in a frozen ocean, especially during a full global glaciation, where marine photosynthesis would be very limited. After the collapse of the Larsen B ice shelf in Antarctica, researchers found evidence of recent chemosynthetic communities, made up of microbial mats and vesicomyid clams (Domack et al., 2005). These methane seeps provide an alternative energy source for sub‐ice shelf communities. However, they were examined 5 years after the ice‐shelf collapse, and whereas microbial assemblages were dominated by anaerobic methanotrophic archaea (Niemann et al., 2009), no live bivalves were found, only empty shells. Hydrothermal vents and cold seeps have also been discovered in the Antarctic deep sea and are capable of supporting high densities of life (Rogers & Linse, 2014). These black smokers, with temperatures up to 382.8°C and diffuse venting, support an abundance of life and are dominated by vast aggregations the yeti crab (*Kiwa tyleri*) exceeding 700 individuals m^−2^, stalked barnacles, limpets, gastropods, anemones, and a predatory sea star (Rogers & Linse, 2014). The hypothesis that many early animals existed either partly or purely through chemosynthetic symbiosis (Dufour & McIlroy, 2017; McMenamin, 1986) would support the idea that a chemosynthetic community could have survived the Neoproterozoic glaciations. As with limited oxygen at this time, sulphur is believed to have been insufficient within the water column to support widespread chemosynthesis (Canfield et al., 2007), although this would not preclude localised hotspots as seen in the deep sea today. Evidence for Cryogenian hydrothermal vent systems include the Dantango Mn deposit (Yu et al., 2016). However, the spatial distribution of the later Ediacaran biota is unrelated to hydrothermal systems (Flude & Narbonne, 2008).

### 2.3 The deep sea

Antarctic bottom water is a globally important feature of a glaciated Antarctic and is formed by surface waters cooling below the ice shelves and in polynyas during the formation of sea ice (Ohshima et al., 2013; Orsi et al., 1999) (Figure 2). This dense, highly saline, oxygen‐rich water rapidly sinks, ventilating the deep sea as it flows northward into the world's ocean basins (Figure 2). This interaction between the ocean and the cryosphere is an important driver of global ocean circulation and survival of aerobic life in the deep sea (Ferrari et al., 2014).

Although atmospheric oxygen was generally low throughout the Neoproterozoic, and potentially into the Cambrian, marine glacial processes and turnover could significantly increase the transfer of gases between the atmosphere and the deep sea (Gordon, 2001). This sinking of heavy, saline, oxygenated water cooled by ice and ice formation could have significantly contributed to the post‐Gaskiers deep‐sea oceanic oxygenation (cf. Ref. Canfield et al., 2007; Figure 1). Furthermore, such sinking of even slightly oxygenated surface water or subglacial meltwater (Lechte et al., 2019) has the potential to create oxic deep‐sea refugia, such as regions beneath polynyas (Figure 2). These theoretical deep sea oxic refugia would likely have a seasonal supply of sinking food from above, as well as from the food banks (in the form of POC/plankton) built up in the sediments during the summer blooms (Fillinger et al., 2013; Smith et al., 2012). In modern Antarctica this benthic‐pelagic coupling serves to dampen the “boom and bust” of seasonal food availability, sustaining benthic organisms over the winter in high latitudes (Smith et al., 2012). Such deep water areas might have provided a refuge from which deep‐sea benthic life may have persisted during the Ediacaran. This oxygenated water can be driven under the ice shelves by currents, with the potential for the creation of further sheltered refugia. Understanding the feasibility of such under‐ice communities would require further modelling of Neoproterozoic global currents but could potentially provide a key refugia for animal life during glacial events.

The Cryogenian biosphere showed stable modelled low atmospheric O_2_ and relatively high seafloor anoxia (Tostevin & Mills, 2020). This relative stability continued through to the Ediacaran where it was punctuated by surface and later deeper ocean oxygenation events (Figure 1; Shields‐Zhou & Och, 2011, Wood et al., 2019), followed by a further decrease in oxygen into the Cambrian (Tostevin & Mills, 2020). Seafloor anoxia levels are thought to have been similar during the early Cambrian to those of the Neoproterozoic (Tostevin & Mills, 2020), but this remains uncertain because the trend is not fully resolved (Rooney et al., 2020; Wood et al., 2019). If correct, this trend would imply that benthic oxygen levels during widespread glaciations (snowball and slushball Earths) would not have been the key limiting factor for the earlier evolution of metazoans (Sperling et al., 2015). Early animals may also have been more tolerant of anoxic and hypoxic environments, requiring far lower oxygen levels than later taxa (Danovaro et al., 2010; Mills et al., 2014). However, on a local scale there is evidence of oxygen oases linked to photosynthetic mats in Neoproterozoic anoxic aquatic environments (Wang et al., 2021) and there is evidence that photosynthesis continued during snowball Earths (Corsetti et al., 2006; Johnson et al., 2017; Martin et al., 2008; Narbonne, 2005; Olcott et al., 2005). Oxygenated subglacial meltwater from the underside of the floating ice cover and ice shelves may have supported aerobic marine habitats during snowball and slushball glaciations (Lechte et al., 2019).

## 3 IMPACTS OF GLACIATION ON HABITATS, PRESERVATION, AND MACROEVOLUTIONARY PATTERNS

Although many icy environments today provide refuge and habitats for specialist organisms, the ice itself can be a highly destructive force within polar marine ecosystems. These effects influence life differently at different depths, with the shallows bearing the brunt of their impact (Smale et al., 2008). During recent glacial periods these impacts are known to have been more severe and have important implications for the habitability, preservation or even existence of shallow water habitats during snowball or slushball Earths.

Shallow water environments around Antarctica today are highly disturbed and rare, due to the presence of ice sheets and shelves, glacial scouring and isostatic depression (Griffiths, 2010). Although rarer than elsewhere in the world, very shallow habitats (<30 m depth) are at risk from anchor ice, brash ice, and small icebergs. Anchor ice forms on the seafloor and is believed to play an important role in subtidal zonation by damaging or killing many sessile organisms as it forms on or around them (Dayton et al., 1969). As it forms, anchor ice can even dislodge animals from the seafloor and float them to the surface. Brash ice is an accumulation of floating ice, made up of fragments of other forms of ice, not more than two metres across. Brash ice scours the intertidal and immediate subtidal zone as it is moved by the wind and tides (Waller et al., 2006).

Iceberg scouring is a key factor that affects Antarctic sea floor communities at depths of less than 25 m (Robinson et al., 2020; Smale et al., 2007), and such scouring can reach down to around 500 m depth (Peck et al., 1999). Grounded icebergs can plough up the seafloor, killing up to 100% of the fauna in their path (Peck et al., 1999). Iceberg scouring increases as an area deglaciates (Barnes, 2017). During and after global glacial events, it is possible that most shallow water environments will have been subjected to frequent and severe iceberg disturbance, which may have deterred shallow water fauna from becoming established and/or may have destroyed evidence of their existence (Gerdes et al., 2008). Although iceberg scour has a catastrophic effect where they come into contact with the benthos, they can also benefit benthic communities that are beyond the reach of the keel of the berg by increasing overall food availability. The presence of icebergs has a positive impact on the planktonic primary productivity of glacial regions, with the input and dispersal of terrigenous nutrients and trace elements during melting, increasing phytoplankton biomass by up to a third (Schwarz & Schodlok, 2009). The giant icebergs generated by the LGM, and Neoproterozoic glaciations would have had even more impact, with icebergs >18 km in length substantially enhancing chlorophyll levels over a radius of 4–10 times the iceberg's length (Duprat et al., 2016).

During the LGM (a glacial period of a much lesser extent compared to the Neoproterozoic glaciations), most of the Antarctic continental shelf is believed to have been covered in thick ice sheets extending almost out to the shelf break (Post et al., 2014), likely covering all continental shelf habitats shallower than 600 m. The expansion of these ice sheets across the continental shelf during multiple recent glacial periods would have destroyed the shelf habitats and any evidence of the fauna present (Thatje et al., 2005). The repeated ploughing of the Antarctic continental shelf by glacial events has severely restricted the shallow water fossil record over recent glacial cycles (Convey et al., 2008), limiting it to exceptional locations protected from the ice or material deposited since the LGM.

The ice sheets of the LGM did not cover the entire Antarctic shelf. Evidence suggests that the most northerly areas of the western Ross Sea, Prydz Bay, a location in George V Land, the Bransfield Strait on the northern tip of the Antarctic Peninsula and potentially parts of the overdeepened Ronne and Filchner Troughs, in the Weddell Sea, would all have been free of grounded ice (Larter et al., 2012), and served as potential refugia for life. The slope and deep sea areas would have been least directly impacted by the expanded ice sheets of the LGM, acting as potential refugia for benthic species able to expand their bathymetric range.

This ploughing could explain the broad‐scale diversity patterns of Ediacaran macrofossils which show a trend from deep water, high‐latitude localities immediately post‐Gaskiers to shallower water and lower latitudes over time (Boag et al., 2016; Grazhdankin, 2014; Waggoner, 2003). However, post‐Gaskiers protist and macroalgae groups are found in shallow water, low‐latitude sites (Boddy et al., 2022) demonstrating that macroscopic life does have at least some preservation potential for these potentially ploughed sites. Furthermore, these macroalgal and protist groups do not show significant differences between low and high palaeolatitudes (Boddy et al., 2022). In contrast, Frondomorphs (soft‐bodied Ediacaran organisms that are sessile with a frond attached to the seafloor) have a significant high‐ to low‐latitude radiation (Boddy et al., 2022), implying that a macro‐evolutionary scale process, rather than simple dispersal and recolonisation, could be responsible.

Preservation potential of Antarctic slope benthic communities is further reduced because the Antarctic slope can be a harsh and unstable environment, with turbidity flows and a substrate made up of glacial debris also making the substrate hard to recolonise by organisms used to low sedimentation rates (Arthur & Garrison, 1986). Further refugia could have come from the freezing of microorganisms and benthic larvae within sea ice (Schnack‐Schiel et al., 2001) or benthic sediments (Jiang et al., 2012), with subsequent thawing and recolonisation of the seafloor. With glacial expansion, animals can migrate to the slope or the deep sea (Brandt, 1992; Brey et al., 1996; Zinsmeister & Feldmann, 1984), find refuge beneath some floating ice shelves, or in areas where grounded ice failed to reach the edge of the continental shelf (Post et al., 2014). With an extreme global glaciation, such as a snowball or partial snowball Earth, these effects would be even more severe, and shallow water habitats, along with the benthos, would have been decimated. If pre‐Gaskiers shallow water benthic animals existed, then during a glacial period, ice would have reached the sea floor in most shelf areas, leaving only rare refugia or the deep‐sea habitable (cf. Post et al., 2014). As the ice retreated, recolonisation of the shallows could have occurred via migration or the release of larvae trapped in ice. However, scouring by sea ice and icebergs may well have continued in shallow water for some time. Further compromising recolonisation were the relatively limited dispersal abilities of Frondomorphs (compared with modern benthos [Mitchell et al., 2015, 2019]). One such example could be the Lantian biota, which is only known in a single region (Yuan et al., 2011) likely prior to the Gaskiers slushball glaciation, increasing the likelihood of extinction and/or demonstrating the rarity of shallow water fossils in a glacial world. Therefore, it is plausible that glacial scouring (Gutt et al., 1996) may explain why non‐algal Lantian taxa are not found post‐Gaskiers.

The shelf would have been similarly impacted by grounded ice during widespread glaciations. Any potential benthic metazoan life would have to have existed in isolated refugia or the deep sea. Although the destructive force and harsh environments of a glacial world can be thought of as inhospitable to life, they can also play a role in driving diversification and evolution. Clarke and Crame (1992) suggested that the fragmentation of ranges by glacial expansion in Antarctica, and subsequent recolonisation of shelf waters in interglacial periods, would have been a mechanism for both extinction and speciation. The glacial–interglacial events of the last 15 million years resulted in a divergence and differentiation between disparate populations in isolated shallower refugia or the slope and deep sea and later between the fauna recolonising the shallows and those inhabiting the deep. Population bottlenecks and cryptic speciation events (Barnes & Kuklinski, 2010; Post et al., 2014) contributed to the so‐called “diversity pump” (Clarke & Crame, 1992).

The idea that the origination and radiation of metazoan life resulted from the succession of snowball and slushball Earths is not a new concept (Hoffman et al., 1998). Radiations driven by the glaciations acting as a source of selective pressure on any life present, would result in environmentally driven genetic bottlenecks and expansions (Hoffman et al., 1998). It is believed that strong greenhouse conditions, driven by high atmospheric CO_2_ concentrations, followed the Cryogenian snowball Earths (Ramme & Marotzke, 2022). However, modelled temperatures and rapid destratification of the ocean suggest that the surface temperatures and conditions would not be as harmful to potential metazoans as previously thought (Ramme & Marotzke, 2022). The extreme environmental conditions and physically separated populations during the Cryogenian and Ediacaran may have been a trigger for and accelerated the pace of early metazoan evolution and diversification. This switching between patchy or isolated glacial refugia during snowball Earths (driven by local oxygen and food oases) and more favorable greenhouse environments, including surface and deep water oxygenation events (Banerjee et al., 2022; Shields‐Zhou & Och, 2011), might have mirrored the recent glacial cycle in Antarctica, only on a much larger scale.

## 4 CONCLUSIONS

The terminal Neoproterozoic, after the Gaskiers slushball glaciation, records the rise of animals in the fossil record, and the transformation of the biosphere from one dominated by microbial life, to one dominated by animals. However, molecular clocks date the origin of animals to hundreds of millions of years earlier, requiring animal life to have survived a period punctuated by global glacial events. The modern‐day cryosphere has a significant impact on the Antarctic biosphere, and so similar processes are likely to have impacted the origins and early evolution of animals. Therefore, by considering modern polar analogues there is potential to gain insights into where and how animals might have persisted during these glacial events.

These polar analogues for early animal survival also have potential utility when considering the search for habitable environments beyond the Earth. For example, three of Jupiter's moons Ganymede, Callisto and Europa and Enceladus from Saturn are thought to be “snowball” moons with potential liquid water underneath, with tantalising hints of potential microbial life and/or hydrothermal vents (Paganini et al., 2020; Postberg et al., 2011). An early, glacial inception of life on Earth and more generally the ideas discussed within this paper could potentially apply to these extra‐terrestrial environments, altering our expectations and understanding of the presence and complexity of potential life elsewhere in the universe.

Here, we argue that life for potential animals living above 500 m depth would have been challenging during Neoproterozoic glaciations due to glacial cover and the impacts of brash ice, anchor ice and icebergs which would have decimated any shallow water benthos. These effects would have limited the feasibility of a widespread shallow water animal origination, persistence, or preservation. Instead, we suggest the majority of animal diversity (if present) would have likely retreated to the deep during a snowball or partial‐snowball Earth, potentially colonising the underside of the ice or deep water refugia, oxygenated via the sinking of cold surface waters formed during sea ice formation or subglacial melting. Furthermore, ice‐induced disturbances may have driven speciation through fragmentation and subsequent recolonization. We conclude that to elucidate the origins of animals we need to understand recent polar biology and apply this knowledge back to the Neoproterozoic.

## Data Availability

Data sharing not applicable ‐ no new data generated, or the article describes entirely theoretical research.
